# Poloxomer 188 Has a Deleterious Effect on Dystrophic Skeletal Muscle Function

**DOI:** 10.1371/journal.pone.0091221

**Published:** 2014-03-18

**Authors:** Rebecca L. Terry, Hannah M. Kaneb, Dominic J. Wells

**Affiliations:** 1 Department of Comparative Biomedical Sciences, Royal Veterinary College, Royal College Street, London, United Kingdom; 2 Department of Cellular and Molecular Neuroscience, Imperial College, Burlington Danes Building, Hammersmith Hospital, London, United Kingdom; The Hospital for Sick Children, Canada

## Abstract

Duchenne muscular dystrophy (DMD) is an X-linked, fatal muscle wasting disease for which there is currently no cure and limited palliative treatments. Poloxomer 188 (P188) is a tri-block copolymer that has been proposed as a potential treatment for cardiomyopathy in DMD patients. Despite the reported beneficial effects of P188 on dystrophic cardiac muscle function, the effects of P188 on dystrophic skeletal muscle function are relatively unknown. *Mdx* mice were injected intraperitoneally with 460 mg/kg or 30 mg/kg P188 dissolved in saline, or saline alone (control). The effect of single-dose and 2-week daily treatment was assessed using a muscle function test on the *Tibialis Anterior* (TA) muscle *in situ* in anaesthetised mice. The test comprises a warm up, measurement of the force-frequency relationship and a series of eccentric contractions with a 10% stretch that have previously been shown to cause a drop in maximum force in mdx mice. After 2 weeks of P188 treatment at either 30 or 460 mg/kg/day the drop in maximum force produced following eccentric contractions was significantly greater than that seen in saline treated control mice (P = 0.0001). Two week P188 treatment at either dose did not significantly change the force-frequency relationship or maximum isometric specific force produced by the TA muscle. In conclusion P188 treatment increases susceptibility to contraction-induced injury following eccentric contractions in dystrophic skeletal muscle and hence its suitability as a potential therapeutic for DMD should be reconsidered.

## Introduction

Duchenne muscular dystrophy (DMD) is an X-linked, fatal muscle wasting disease for which there is currently no cure and limited palliative treatments. DMD is caused by the loss of functional dystrophin protein, primarily resulting from frame-shifting mutations in the dystrophin gene [1]. Dystrophin, and its dystrophin-associated glycoprotein complex connect the extracellular matrix to the cytoskeleton of myofibres and anchor subsarcolemmal neuronal nitric oxide synthase (nNOS) [2]. The lack of dystrophin in DMD muscle increases the permeability of the sarcolemma, particularly during lengthening or “eccentric” contractions [3] such as those performed while walking down stairs. The mechanism behind this is controversial. Traditionally it was thought that the lack of sarcolemmal stabilisation during muscle contractions resulted in membrane tearing causing an influx of calcium [4], [5]. However it has since been demonstrated that stretching dystrophic muscle fibres opens stretch-activated calcium channels, which remain closed in normal muscle [6], [7], and that blocking these channels pharmacologically reduces dystrophic pathology following a period of eccentric contractions [7].

Despite this the “membrane sealant” Poloxamer-188 has received attention as a potential therapeutic in DMD [8]–[10]. Poloxamer-188 (P188, or Pluronic F68) is a non-ionic tri-block copolymer of poly(ethylene oxide)_80_-poly(propylene oxide)_27_-poly(ethylene oxide)_80_ with an approximate molecular weight of 8.4 kDa [11]. P188 is amphiphilic, with a hydrophobic midsection that inserts into areas of low membrane tension, such as areas of membrane damage [12]. P188 has been shown to act as a membrane sealant following a variety of types of injury. In neural tissue P188 was shown to reduce brain damage following ischemia/reperfusion injury *in vivo* [13]. In skeletal muscle intravenous P188 reduced tissue oedema and inflammation in *flexor digitorum brevis* muscles of rats following electric shock [14] and improved myocyte survival after exposure to ionising radiation *in vitro* [15].

In dystrophic muscle P188 has primarily been studied as a potential cardioprotective agent with beneficial effects of systemically administered P188 reported in both dystrophic mouse (*mdx*) and dog (Golden Retriever Muscular Dystrophy (GRMD)) models [8]–[10]. In *mdx* mice both single dose and 2-week treatment with P188 improved left ventricular function and increased survival after challenges with cardiac stimulants [8], [10]. In the more severely affected GRMD dog an 8-week constant rate infusion of P188 resulted in reduced myocardial fibrosis and left ventricular remodelling (Townsend et al. 2010). To date the effect of P188 on skeletal muscle function has only been studied *in vitro*. A modest reduction in isometric contraction-induced force decline was seen in *mdx* lumbrical (toe) muscles bathed in P188 *in vitro* [16] and 2 or 4 weeks of systemic P188 treatment had no effect on specific force production by excised *extensor digitorum longus* (EDL) muscles [10]. However very little is known about the effect of P188 on skeletal muscle function *in vivo*.

P188 has previously reached phase III clinical trials as a rheologic agent for the treatment of sickle cell crisis [17], [18]. Similar compounds have also been used to facilitate delivery of chemotherapeutics to tumours [19]. In this case poloxamer compounds (or “pluronics”) are used to disrupt the plasma membrane of tumour cells by disturbing lipid packing [20], increasing phospholipid flip flop [21], and causing ATP depletion [22]. This approach has also been used to enhance gene delivery to several tissues including skeletal muscle [23]. Therefore the addition of P188 to the sarcolemma is unlikely to be a benign event. The hypothesis for this study was therefore that P188 would have a deleterious effect on skeletal muscle function *in vivo*, particularly when the membrane is subjected to mechanical stress.

## Materials and Methods

### Ethical approval

All animal experiments were carried out under license from the Home Office (UK) in accordance with The Animals (Scientific Procedures) Act 1986 and were approved by Royal Veterinary College ethical committee. All mice were housed in a minimal disease facility and had *ad libitum* access to food and water.

### Animals

Homozygous female *mdx* mice were used for muscle function studies and male *mdx* mice were used for the tail vein bleeding time assay. All mice in the P188 study were 9–11 months old. Littermates (and cagemates) were distributed across the three treatment groups with 7–9 mice in each group in a randomised block design. In addition, data is provided from female 6-month old *mdx* (n = 5) and C57BL/10 mice (n = 7) to illustrate the difference in contraction-induced injury between *mdx* mice and their background wild-type strain.

### Drug treatment for muscle electrophysiology studies

P188 was dissolved in sterile saline to form a 100 mg/ml stock solution. Treated *mdx* mice either received 460 mg/kg [10], [14] P188 or 30 mg/kg P188 [18]. Control mice received saline alone. All solutions were made up to 0.2 ml with saline and given by intraperitoneal (IP) injection. Peak plasma concentrations of P188 have been reported to occur between 20 and 120 mins after IP injection [24].

For the single-dose study mice were weighed on the day of surgery and injected with either P188 or saline 15 minutes prior to the induction of anaesthesia. The eccentric contraction protocol therefore started approximately 90 minutes after drug administration. For the 2-week dose study mice were injected daily at the same time in the morning for 2 weeks. Mice were weighed twice a week throughout treatment. Muscle function measurements were made in the afternoon to try to avoid any acute effects of treatment that may have occurred during the peak plasma concentrations.

### In situ TA muscle function testing

Muscle force measurements were made using an *in vivo* protocol that has been described previously [25], [26]. Mice were deeply anaesthetised with fentanyl/fluanisone (Hypnorm) and midazolam (Hypnovel) given by intraperitoneal (IP) injection. Solutions of Hypnorm, water for injection and Hypnovel were made in a volume ratio of 1∶2∶1 and administered at 8 µl/g. Anaesthesia was maintained with further IP injections so that the animal remained insensitive to toe pinch and surgical stimuli. Surgical preparation of the TA tendon and the common peroneal branch of the sciatic nerve and subsequent data acquisition were conducted with the animal on an electric heat pad under a light source to maintain normothermia. Exposed tissues were kept moist by frequent irrigation with warm saline.

**Figure 1:**
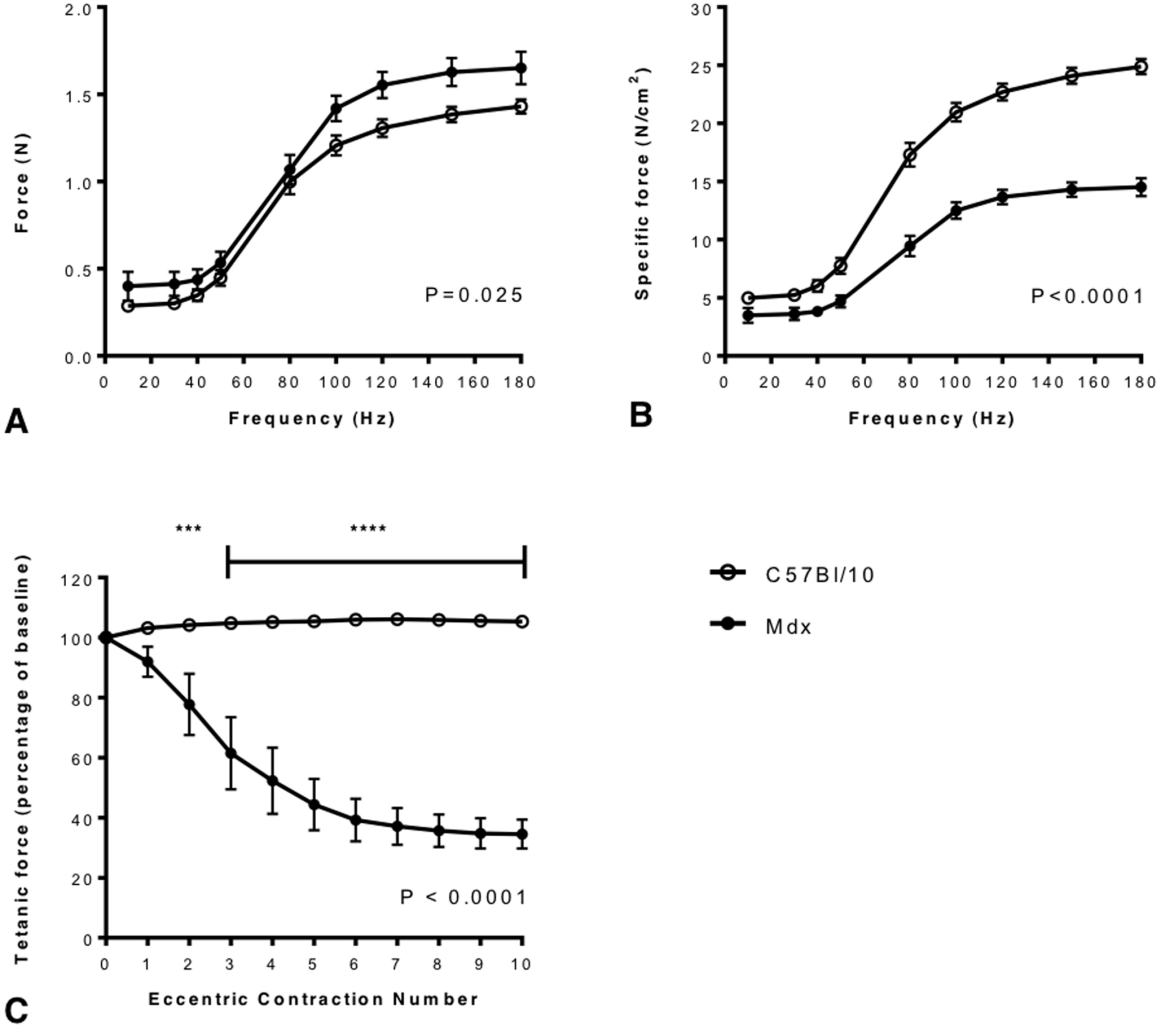
Typical force frequency relationships and response to eccentric exercise in *mdx* and C57BL/10 mice. TA muscles from anaesthetised female 6 month old *mdx* (n = 5) and C57BL/10 mice (n = 7) underwent a series of isometric contractions *in situ* induced by stimulation of the common peroneal nerve using 1.3v at 10, 30, 40, 50, 80, 100, 120, 150 and 180 Hz. *Mdx* mice were significantly stronger than C57Bl/10 mice when absolute force (N) is compared at each frequency (A). However C56Bl/10 mice were significantly stronger than *mdx* when force was normalised to TA muscle cross sectional area (Specific force N/cm^2^; B). Following the isometric contraction protocol a series of eccentric contractions of the TA *in situ* was induced by stimulation of the common peroneal nerve at 120 Hz and simultaneously stretching the TA by 10% of its optimum length. Tetanic force was measured prior to each stretch and expressed as a percentage of the baseline force recorded prior to the first stretch (C). No drop in force occurred in C57Bl/10 mice after 10 eccentric contractions, however the force produced by *mdx* mice was significantly weaker than C57Bl/10s from contraction 2 onwards, dropping to 35% of baseline by contraction 10, ***P<0.001, **** P<0.0001. Error bars represent SEM.

After an initial warm up protocol of 5 submaximal stimulations 1 minute apart the muscle's optimum length (L_o_) was determined by measuring a series of twitches at increasing resting tensions. The resting tension that produced the strongest twitch was then used throughout the protocol and this twitch was recorded and used for measurement of time to peak tension (TTPT, ms) and half-relaxation time (ms). A series of stimulations at 10, 30, 40, 50, 80, 100, 120, 150 and 180 Hz were delivered 1 minute apart to measure the force-frequency relationship. The maximum isometric tetanic force (P_o_) was determined from the plateau of the force-frequency curve. Muscle length was measured using digital calipers. Total functional muscle fibre cross-sectional area (CSA in cm^2^) was calculated by dividing the muscle weight in grams by the product of the TA fibre length (Lf; cm)×the density of mammalian skeletal muscle (1.06 g/cm^3^). Because the TA is a pennate muscle, Lf is 60% of the measured optimal length [27]. Specific force (N/cm^2^) was calculated by dividing the absolute force (N) at each stimulation frequency by TA muscle cross-sectional area. Specific force was plotted against frequency and curves are compared using a Two-way Repeated Measures Analysis of Variance (2-way RM ANOVA) with Tukey's *post-hoc* comparison. A representative graph of the force-frequency relationship in female C57BL/10 and *mdx* mice is provided in Figure 1A.

The muscle was then allowed to rest for 5 minutes before the eccentric contraction protocol was initiated. This protocol uses a physiologically relevant stretch of 10% of L_0_ that does not cause a drop in isometric force in wild-type mice (Figure 1B and [25]) in contrast to greater stretches of 15% over the same time period [26]. A tetanic contraction was induced using a stimulus of 120 Hz for 700 ms. During the last 200 ms of this contraction the muscle was stretched by 10% of L_o_ at a velocity of 0.5 L_o_ s^−1^ and then relaxed at −0.5 L_o_s^−1^ following the end of the stimulus. The muscle was subjected to 10 eccentric contractions, delivered 2 minutes apart to avoid the confounding effect of muscle fatigue. The mouse was then euthanised by cervical dislocation and the stimulated muscle was immediately carefully removed and weighed. The isometric force recorded prior to the first stretch was used as a baseline. The isometric force prior to each subsequent stretch was measured and expressed as a percentage of this baseline force. Isometric force was plotted against eccentric contraction number and curves were compared using a 2-way RM ANOVA with Tukey's *post-hoc* comparison. Mice that did not complete the full protocol (either due to technical difficulties or death under anaesthesia) were excluded prior to statistical analysis.

### Preparation of muscle sections

Mice were dissected immediately after euthanasia. Muscles were mounted on cork discs, coated with Cryo-M-Bed and snap frozen in liquid nitrogen cooled isopentane then stored at −80°C until sectioning. Following a period of equilibration muscles were cut in serial transverse, 10 µm thick sections using a Bright cryostat at −25°C. Sections were collected onto 12 Superfrost™ slides every 300 µm along the whole length of the muscle. Slides were allowed to dry then stored at −80°C until staining.

### Staining of muscle sections

Slides were allowed to dry for 30 minutes before staining. For all stains all 8–12 sections on each slide were stained thereby allowing analysis of up to 8–12 levels along the length of the muscle.

H&E staining was used to assess muscle histopathology. Slides were rehydrated in distilled water, stained for 2 minutes in Harris's Haematoxylin, dipped in acid alcohol, placed in running tap water for 3 minutes, stained for 2 minutes in Eosin then dehydrated through graded alcohols, cleared in xylene and mounted using DPX.

Alizarin red was used to identify increased levels of intra- cellular calcium in exercised and unexercised TA muscle. Sections were fixed in cold acetone for 10 minutes then air-dried. Dried sections were rinsed in distilled water then placed in a filtered alizarin red solution for 1 minute. Slides were blotted, rinsed in acetone for 30 seconds, then rinsed in a 1∶1 solution of acetone∶xylene for 15 seconds before being cleared in xylene and mounted. Fibres containing increased levels of bound calcium stain dark red. The alizarin red solution was made by dissolving 2 g of Alizarin Red S (sodium alizarin sulphonate) in 100 ml distilled water and adjusting to pH 4.2 with hydrochloric acid.

Staining for intracellular IgG was used to indicate increased permeability of the sarcolemma. Intracellular IgG staining was chosen because it yields similar results to measuring Evans Blue Dye uptake but does not require the mice to be injected [28]. Dried slides were rehydrated in phosphate buffered saline containing 0.05% tween 20 (PBST), blocked using an avidin biotin blocking kit (Vector Laboratories, UK) then washed in PBST. Sections were incubated at room temperature with a biotinylated anti-mouse IgG antibody (Mouse on Mouse Kit, Vector Laboratories, UK, diluted 1∶50 in PBST). Sections were then washed and developed using an ABC kit (Vector Laboratories, UK) and metal enhanced DAB (Sigma) staining. Fibres containing intracellular IgG stained dark grey against a pale grey background.

### Quantification of histologic parameters

Slides were evaluated blind to sample identity using a Leica DMLB microscope and Q-Capture software. Muscle pathology was assessed in the largest section from the mid-belly of each TA. The number of alizarin red positive, IgG positive and hypercontracted fibres present in each H & E section were manually counted. Hypercontracted fibres are enlarged, rounded and stain strongly with eosin (hypereosinophilic). In addition a qualitative 5 point scoring system was used to score exercised and unexercised TA muscles based on the level of necrosis, inflammatory cell infiltrate, fibrosis, interstitial oedema and grossly damaged fibres present (Figure S1). Data were analysed using unpaired t-tests or one-way AVOVA and Tukey's post-hoc comparison where appropriate.

### Tail vein bleeding time assay (TVBT)

Mice were injected each morning for 2 weeks, and were weighed twice a week during treatment. On the afternoon of the last dose the mice were anaesthetized with isofluorane and a TVBT assay was performed as previously described [29]. An incision was made over the right lateral tail vein and the tail was placed into a falcon tube of saline, pre-warmed to 37°C. Bleeding was timed from incision to cessation. Mice were then euthanised by cervical dislocation. Data were analysed using a one-way ANOVA and Tukey's post-hoc comparison.

## Results

### Typical force-frequency relationship and response to eccentric exercise in mdx and C57BL/10 mice

During the force-frequency protocol the TA muscles of *mdx* mice produced greater absolute force (N) during isometric contractions than those of C57Bl/10 mice at each frequency (P = 0.025, Figure 1A), however when force was normalised to TA muscle cross sectional area (N/cm^2^) *mdx* TA muscles were markedly weaker than those of C57Bl/10 mice (P<0.0001,Figure 1B). During the eccentric contraction protocol no drop in force output occurred in the TA muscles of C57Bl/10 mice after 10 eccentric contractions, however the force produced by *mdx* mice was weaker than C57Bl/10s from contraction 2 onwards, dropping to 35% of baseline by contraction 10 (P<0.0001, Figure 1C).

### P188 study

There was no significant change in body mass throughout the experiment in any of the treatment groups. There was no difference in mean body mass between treatment groups within the cohorts of female and male mice. All animals completed the treatment with no negative clinical signs.

### Single dose P188 treatment induced a mild increase in specific force

**Figure 2:**
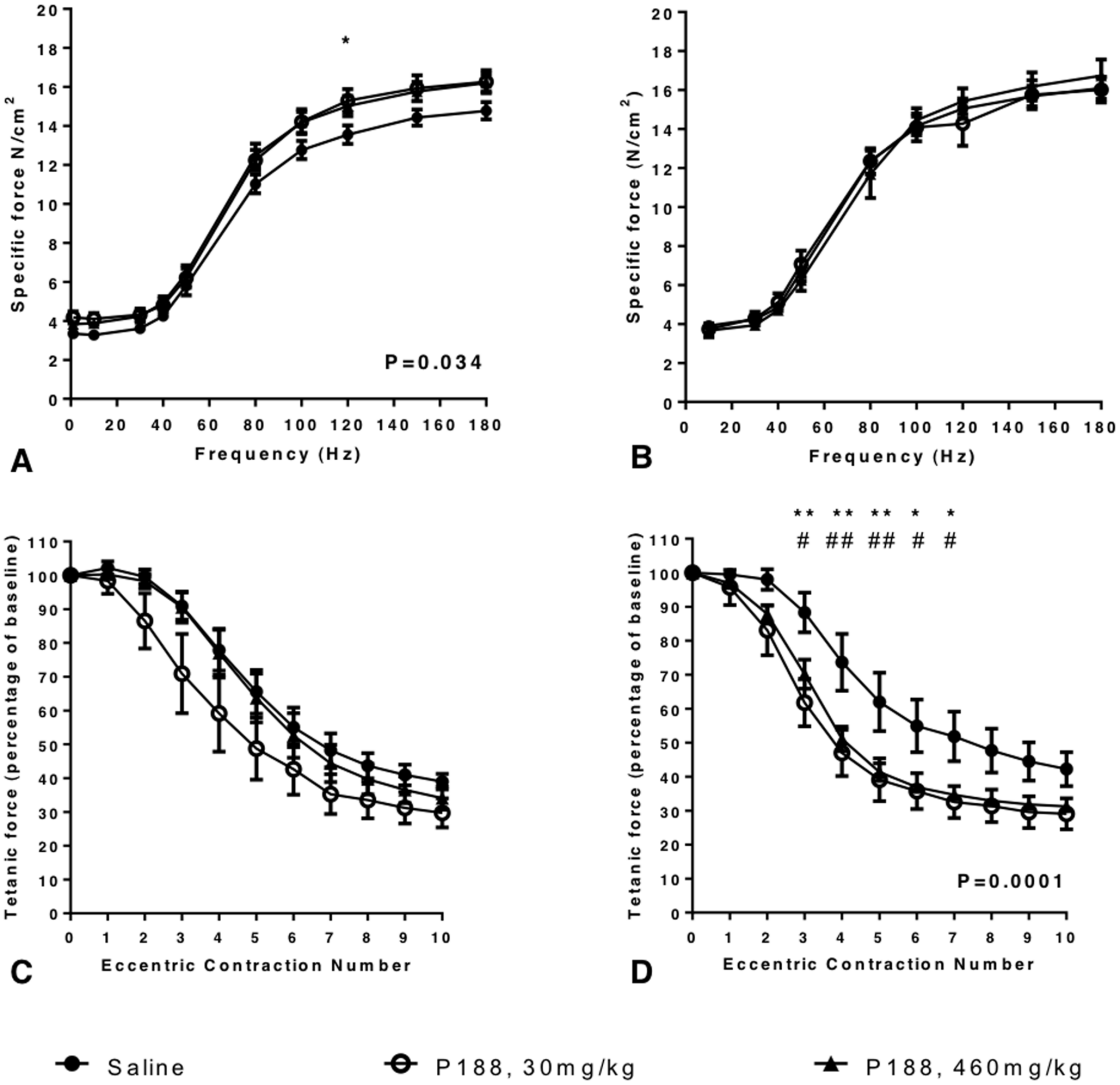
The effect of P188 on TA muscle function. The force frequency relationship was examined in P188-treated 9–11 month old female *mdx* mice and saline-treated controls (n = 6 in each group). TA muscles from anaesthetised *mdx* mice underwent a series of isometric contractions *in situ* induced by stimulation of the common peroneal nerve at 10, 30, 40, 50, 80, 100, 120, 150 and 180 Hz. For mice given a single dose of P188 the force-frequency curves for the TA muscles of P188 treated mice were significantly different from that of saline-treated control mice (P = 0.034, A). Mice treated with 30 mg/kg P188 exhibited higher specific forces than saline-treated mice when the common peroneal nerve was stimulated at 120 Hz (* P<0.05). However after 2 weeks of treatment the force frequency curves were not different (B). In contrast 2 weeks of treatment with P188 at either dose increased the magnitude of the drop in force production by TA muscles during a protocol of 10 eccentric contractions with a 10% length increase (P = 0.0001; D). Post-hoc analysis revealed P188 treated mice were significantly weaker than saline treated controls at contractions 3–7 inclusive. This effect was not seen after a single dose of P188 (C). Tetanic force is expressed as a percentage of baseline isometric force produced prior to the first stretch. * P<0.05 ** P<0.01 saline treated vs P188 30 mg/kg; # P<0.05 ## P<0.01 saline-treated vs P188, 460 mg/kg. Error bars represent SEM.

Over a range of frequencies from 10 to 180 Hz single-dose P188 treatment induced a mild increase in TA specific force (P = 0.03, RM ANOVA). On post-hoc analysis (Tukey's) the 30 mg/kg P188 treatment group were stronger than the saline group when stimulated at 120 Hz (P<0.05) with specific forces of 15.29±0.59 N/cm^2^ and 13.55±0.48 N/cm^2^ respectively (Figure 2A). Maximum specific forces were 14.78±0.44 N/cm^2^ (saline), 16.27±0.59 N/cm^2^ (30 mg/kg P188) and 16.21±0.46 N/cm^2^ (460 mg/kg P188), and were not significantly different. No differences were detected in specific twitch tension, time to peak tension or half relaxation time between the 3 groups (data not shown). After 10 eccentric contractions the mean TA tetanic forces were 39±2.31% and 27.9±4.33% of the first contraction in saline and 30 mg/kg P188 treated mice respectively, however this was not statistically significant (Figure 2C).

### Two-weeks of daily P188 did not change TA muscle specific force

In contrast to the single dose study in which mice were injected 15 minutes prior to surgery, two-weeks of daily P188 treatment did not significantly change the specific force produced by the TA over the same range of frequencies (Figure 2B). Maximum specific forces were 16.74±0.83 N/cm^2^ (saline), 16.02±0.65 N/cm^2^ (30 mg/kg P188) and 16.1±0.56 N/cm^2^ (460 mg/kg P188). No significant differences were detected in specific twitch tension, time to peak tension or half relaxation time between the 3 groups (data not shown).

### Mice treated for two-weeks with either 460 mg/kg or 30 mg/kg P188 exhibited an increased sensitivity to eccentric contractions

After 2 weeks of daily P188 treatment the force produced by the TAs of mice in both 30 mg/kg and 460 mg/kg dose groups during the eccentric contraction protocol was lower than that of saline-treated control mice (P = 0.0001). Specifically, P188 treated mice in both treatment groups were significantly weaker than saline treated mice at contractions 3–7 inclusive. There was no difference in force between mice receiving 30 mg/kg and 460 mg/kg P188 at any contraction number (Figure 2D). After 10 eccentric contractions the TA mean tetanic forces were 42.2±5% (saline), 29.1±4.6% (P188, 30 mg/kg) and 31.3±1% (P188, 460 mg/kg) of the tetanic force at the first contraction.

### P188 treatment improved the histological appearance of unstimulated, but not stimulated TA muscles

**Figure 3:**
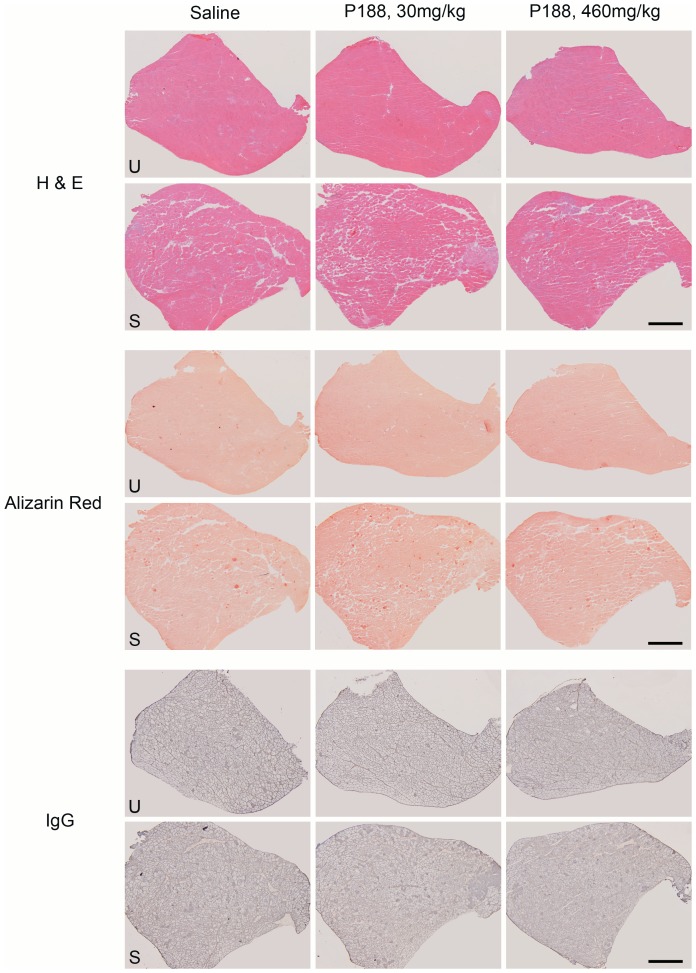
Representative images from unstimulated and stimulated TA muscles from P188-treated and control 9–11 month old female mdx mice. After muscle isometric force measurements and the eccentric contraction protocol were completed mice were immediately euthanised and dissected. The stimulated and unstimulated TA muscles were mounted in the same block, cryosectioned and stained with H&E, Alizarin red and biotinylated anti-mouse IgG antibody followed by nickel-enhanced DAB). Representative low power images of serial sections from a single mouse from each treatment group are shown above. For each stain the upper row is images of the unstimulated TA (U) and the lower row is images of the stimulated TA muscle (S) from the same mouse. While there appears to be a mild reduction in H and E score (Figure S1) in the unstimulated muscle from the 30 mg/kg P188 group this difference is not seen in the stimulated muscles. Similarly there are less IgG positive fibres (dark grey) in the unstimulated muscles of the P188-treated group but not in the stimulated muscles. For all treatment groups stimulated muscles are expanded by marked oedema and contain greater numbers of hypercontracted (densely eosinophilic and rounded), alizarin red positive (bright red) and IgG positive (dark grey) fibres. Scale bars represent 1000 µm.

**Figure 4:**
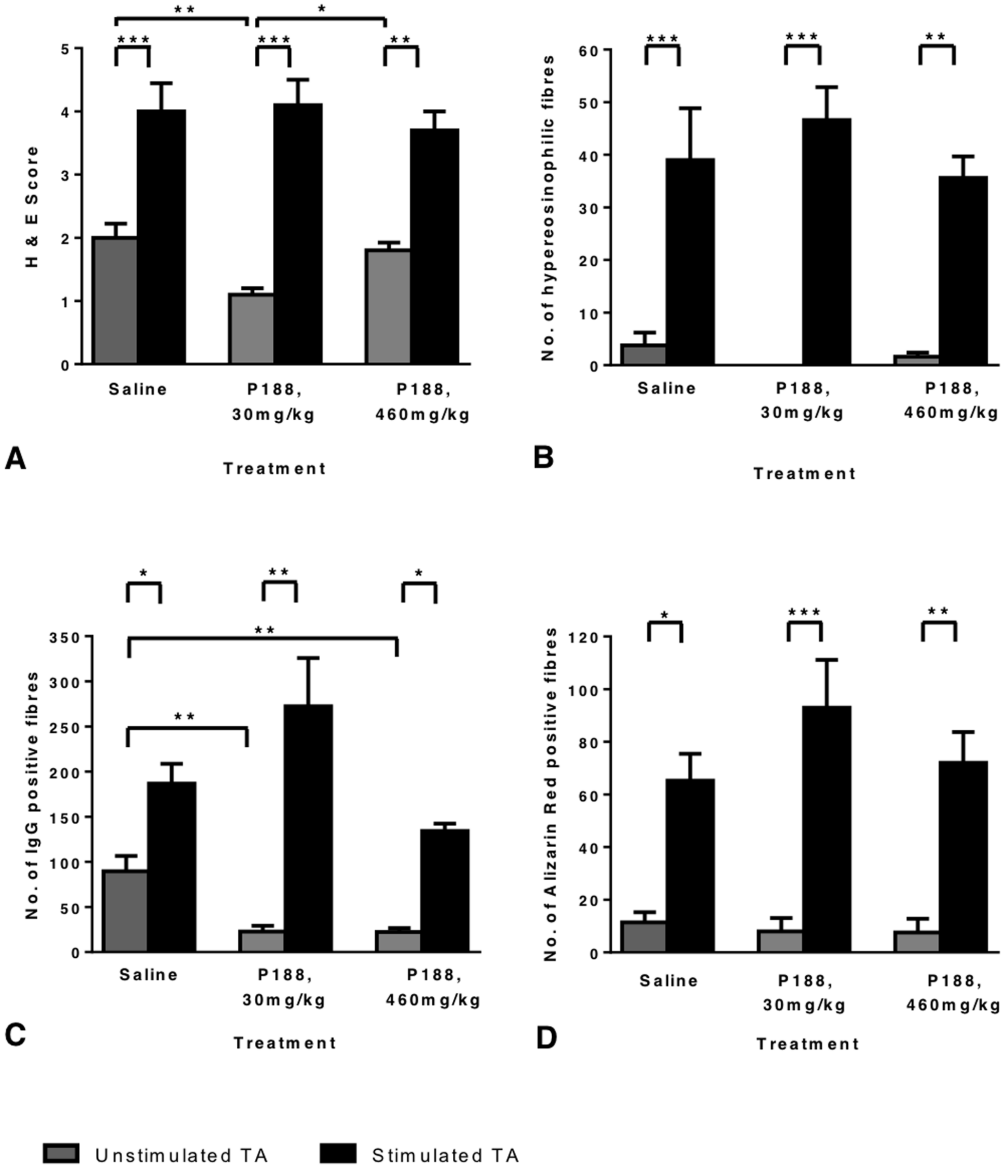
The effect of two weeks of P188 treatment on TA muscle histology. After muscle isometric force measurements and the eccentric contraction protocol measurements were completed mice were immediately euthanised and dissected. Both TA muscles were mounted in the same block, cryosectioned and stained with H&E, Alizarin red or biotinylated anti-mouse IgG antibody followed by nickel-enhanced DAB. Muscle pathology was scored in H & E sections (A) and hypereosinophilic (hypercontracted) fibres were counted (B). The number of IgG positive fibres (C) and AR positive fibres (D) were also counted. All counts and scores were made while blind to sample identity. There was a significant increase in H & E score, hypereosinophilic, IgG and AR positive fibres between un-stimulated (grey bars) and stimulated (black bars) TA muscles in all treatment groups. There was a significant reduction in IgG positive fibres and H& E score in the P188-treated, unstimulated muscles. N = 5, 9–11 month old female *mdx* mice in each treatment group. Error bars represent SEM. * P<0.05, ** P<0.01, *** P<0.001.

H & E sections were scored on a 5-point scale based on the levels of inflammation, oedema and necrosis/regeneration present (Figure S1). Representative images of each treatment group are displayed in Figure 3. Unstimulated TA muscles from the 460 mg/kg and 30 mg/kg P188 groups had a significantly smaller number of fibres with internalised IgG than the saline treated group (Figure 4C), indicating that fewer fibres had sufficient sarcolemmal damage for IgG to cross the membrane. In addition the unstimulated muscles from the 30 mg/kg P188 group had a significantly lower H & E score than the saline treated group (Figure 4A). The H & E score of the 460 mg/kg group was not different from the saline group. The number of Alizarin red positive fibres did not differ across the groups in the unstimulated muscles (Figure 4D).

However P188 did not improve the histological appearance of the stimulated TA muscles. In all treatment groups, stimulated muscles showed a significantly higher number of IgG or Alizarin red positive fibres, and increased H & E score compared to the unstimulated contralateral muscles. The number of hypercontracted, hypereosinophilic, swollen fibres was also significantly higher in stimulated muscles (Figure 4B).

### Tail vein bleeding time

This assay was conducted because some mice appeared to bleed more than expected during surgery. Mice treated with 460 mg/kg P188 for 2 weeks bled longer than mice treated with 30 mg/kg P188 or with saline. The mean bleeding time exhibited by high dose treated mice was 76.07±11.4 s compared to 47.4±2.4 s and 54.9 ±7.2 s of control and low dose treated mice respectively (P<0.05).

## Discussion

The amphiphilic, non-ionic tri-block copolymer P188 has been suggested as a potential therapeutic for DMD due to its ability to insert into the areas of low membrane tension that may occur in damaged, dystrophic membranes. While systemic P188 treatment has been shown to improve cardiac function in *mdx* mice and GRMD dogs the study reported here is the first to document the effects of P188 on dystrophic skeletal muscle function *in situ*, with an intact blood supply. This test removes the confounding effects of respiratory and cardiac function, variable body mass and the motivational state of the mice associated with treadmill running.

In contrast to studies of the P188-treated *mdx* lumbrical muscle [16] or EDL [10] *in vitro*, *in situ* muscle function testing has demonstrated a negative effect of P188. In the current study, treatment with P188 administered IP for 2 weeks did not change the specific force generated by the TA muscle of homozygous female *mdx* mice. Treatment did however reduce the muscles' resistance to contraction-induced injury, resulting in an earlier and greater drop in force during a protocol of 10 eccentric contractions; despite reduced IgG uptake and qualitative H & E scores in the unstimulated muscles of P188-treated mice.

Changes in membrane permeability markers following P188 treatment have been reported in previous studies. In a study in which *mdx* mice received a single IP dose of 600 mg/kg P188 Evans blue dye (EBD) uptake was reduced in unexercised treated mice compared to unexercised controls. However this significant difference was not present after the mice were exercised on a treadmill [24]. In addition, an increase in EBD uptake was noted in the cardiac muscle of P188 treated (460 mg/kg) *mdx* mice compared to untreated controls following an isoprotenolol challenge that induces tachycardia and hypertension [10]. This would suggest that although P188 may produce modest improvements in membrane permeability in resting muscle, it may actually increase membrane permeability under conditions where the membrane is exposed to increased mechanical stress.

In the aforementioned treadmill study mice treated with 600 mg/kg P188 ran shorter distances than placebo treated control animals and were unable to complete the treadmill test. Although this difference was not significant (P = 0.09) it lead to a modification of experimental protocol due to the “adverse effect” of P188 (Quinlan et al. 2006). In light of the results reported here their findings would support the notion that P188 has a deleterious effect to exercising skeletal muscle. Finally, myalgia in leg and back muscles was frequently reported in humans on the higher doses of IV P188 during pharmacokinetic studies [11], [30]. While it is possible that a toxic plasma level may have been reached at the higher doses of 600 mg/kg [24] and 460 mg/kg, negative effects on the TA muscle's response to eccentric contraction were seen in the current study at a dose of 30 mg/kg, at least 15 times lower than the high doses used in previous studies [10], [14], [24].

Mice receiving a single dose of P188 demonstrated a modest, but significant increase in specific force that was not seen in the mice that were treated for two weeks. P188 has been taken to phase III clinical trials as a rheologic agent [17] so it is possible the drug improved perfusion through the skeletal muscle capillary beds. Due to the mislocalisation of nNOS, normal exercised induced hyperemia is inhibited in dystrophic muscle [2]. Therefore it is possible that the increase in specific force following P188 injection is due to improved perfusion and hence improved oxygen delivery to the exercising muscle. This would be expected to be greatest during peak plasma concentrations, which would be expected to occur by 2 hours after IP injection [24].

The rheologic action of P188 is also possibly responsible for the increased bleeding time observed in treated mice, although the drug has also been reported to have anticoagulant properties [31]. During surgery it was felt that P188 treated female mice bled more than control mice. To investigate this observation further, a tail vein bleeding time (TVBT) assay was performed in a second cohort of mice. There was no significant difference in bleeding time between saline and 30 mg/kg groups, however the mean bleeding time of the 460 mg/kg treated group was significantly prolonged. The effect of P188 on haemostasis is particularly relevant to DMD patients because they frequently have to undergo long, complicated spinal surgeries and often suffer fractures [32].

The previously reported beneficial effect of P188 on cardiac output appears to be mediated by an increased compliance of the cardiac muscle allowing increased ventricular filling (preload) and subsequent increase in stroke volume and cardiac output [8], [9]. This increase in compliance may not be beneficial in skeletal muscle undergoing eccentric contractions, such as those occurring during walking downstairs. The altered membrane compliance associated with poloxamer administration has been shown to alter functional activity of membrane proteins and change electrical properties of membranes *in vitro* (Demina et al. 2005). Also while P188 only inserts into areas of low surface tension it is expelled from these areas as membrane tension increases to physiologic levels [12]. Therefore although mechanically plugging a hole at rest, the addition of P188 to a skeletal muscle membrane may actually destabilise it further during increased mechanical stress. Finally it has been demonstrated that P188 can act as a permeabiliser rather than a sealant depending on a delicate balance between adsorption of P188 to the membrane surface and insertion of P188 into the bilayer [33]. Adsorption happens on initial exposure and is responsible for the sealant action however after longer exposure the molecule inserts into the bilayer and increases permeability [33]. Therefore longer-term treatment might conceivably increase sarcolemmal permeability further.

In conclusion, in contrast to its beneficial effects on dystrophic cardiac function, systemically administered P188 increases contraction-induced injury in skeletal muscle, even at a low dose and also increases bleeding time. Taken together, these results question the use of P188 as a therapeutic in DMD, particularly in patients that are still ambulant.

## Supporting Information

Figure S1 **Representative images of mdx TA muscles with H & E scores of 1–5.** H & E stained cryosections of TA muscles from 6-month old WT and *mdx* mice. Muscles from C57Bl/10 mice (WT) show no pathology and have peripherally located nuclei. *Mdx* muscles given a score of 1 exhibit centrally located nuclei (CN) and fibre size variation but minimal necrosis. A score of 2 is given if muscle has the features of score 1 but with signs of active necrosis (N) and regeneration (R). Score 3 is given if this regeneration and necrosis is extensive, hypercontracted fibres are present but oedema is mild. Muscles receive score 4 if oedema (O) is moderate with numerous hypercontracted fibres (H) and muscle with score 5 exhibit all the features of scores 1–4 and extensive oedema and grossly disrupted fibres (D). Muscles exhibiting features intermediate between two scores were given an additional 0.5. Scale bars represent 100 µm.
(TIFF)
