# Correction: Poloxomer 188 Has a Deleterious Effect on Dystrophic Skeletal Muscle Function

**DOI:** 10.1371/journal.pone.0119252

**Published:** 2015-03-02

**Authors:** The PLOS ONE Staff

The following information is missing from the Competing Interest section: The authors have the following competing interests to declare: Dr. Wells is on the Scientific Advisory Board of Akashi Therapeutics (was Dart Therapeutics), a company developing treatments for Duchenne muscular dystrophy. Dr. Wells is also a member of the Treat-NMD Advisory Committee for Therapeutics that provides confidential guidance on the translation and development path of therapeutics programs in rare neuromuscular diseases. This does not alter the authors' adherence to all PLOS ONE policies on sharing data and materials.

In the title and abstract of this article, the word “Poloxomer” should be “Poloxamer.” The correct title is “Poloxamer 188 Has a Deleterious Effect on Dystrophic Skeletal Muscle Function.” The correct citation is: Terry RL, Kaneb HM, Wells DJ (2014) Poloxamer 188 Has a Deleterious Effect on Dystrophic Skeletal Muscle Function. PLoS ONE 9(3): e91221. doi:10.1371/journal.pone.0091221
