# Supplementary figures and images for: Poloxomer 188 Has a Deleterious Effect on Dystrophic Skeletal Muscle Function
Source: PLoS One. 2014 Mar 18;9(3):e91221. doi: 10.1371/journal.pone.0091221 (PMC3958340; doi:10.1371/journal.pone.0091221)

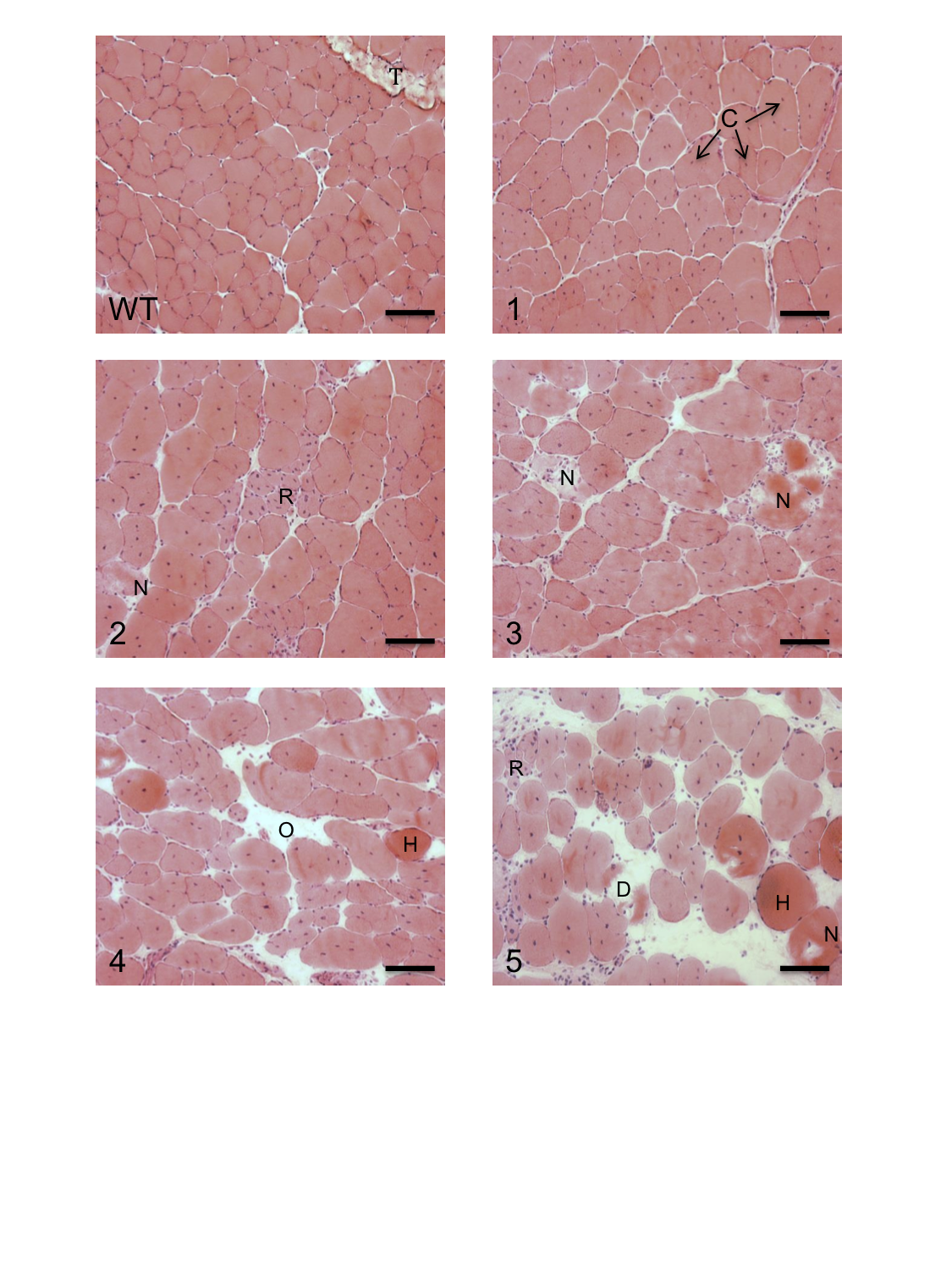

Supplement: Figure S1 — Representative images of mdx TA muscles with H & E scores of 1–5. H & E stained cryosections of TA muscles from 6-month old WT and mdx mice. Muscles from C57Bl/10 mice (WT) show no pathology and have peripherally located nuclei. Mdx muscles given a score of 1 exhibit centrally located nuclei (CN) and fibre size variation but minimal necrosis. A score of 2 is given if muscle has the features of score 1 but with signs of active necrosis (N) and regeneration (R). Score 3 is given if this regeneration and necrosis is extensive, hypercontracted fibres are present but oedema is mild. Muscles receive score 4 if oedema (O) is moderate with numerous hypercontracted fibres (H) and muscle with score 5 exhibit all the features of scores 1–4 and extensive oedema and grossly disrupted fibres (D). Muscles exhibiting features intermediate between two scores were given an additional 0.5. Scale bars represent 100 µm. (TIFF) [file pone.0091221.s001.tiff]
